# Overview and Comparison of PLA Filaments Commercially Available in Europe for FFF Technology

**DOI:** 10.3390/polym15143065

**Published:** 2023-07-17

**Authors:** Vladislav Andronov, Libor Beránek, Vojtěch Krůta, Lucie Hlavůňková, Zdeňka Jeníková

**Affiliations:** 1 Department of Machining, Process Planning and Metrology, Faculty of Mechanical Engineering, The Czech Technical University in Prague, 160 00 Prague, Czech Republic; libor.beranek@fs.cvut.cz (L.B.); vojtech.kruta@fs.cvut.cz (V.K.); lucie.hlavunkova@fs.cvut.cz (L.H.); 2 Department of Materials Engineering, Faculty of Mechanical Engineering, The Czech Technical University in Prague, 160 00 Prague, Czech Republic; zdenka.jenikova@fs.cvut.cz

**Keywords:** 3D printing, additive manufacturing, PLA, filament comparison, FFF, FDM

## Abstract

This study presents a comprehensive techno-economic analysis of PLA materials for fused filament fabrication (FFF) from eight European manufacturers. The comparison involved rigorous experimental assessments of the mechanical properties, dimensional accuracy, and print quality using standardized methods and equipment such as tensile and CT testing. What makes this study unique is the consistent methodology applied, considering factors such as material color, printing temperature, printing orientation, filament diameter, and printer selection, to ensure meaningful and reliable results. Contrary to the common belief that a higher price implies better quality, the study revealed that the second cheapest PLA material achieved the best overall performance within the methodology employed. The study also confirmed certain observations, such as the influence of printing orientation and geometry on dimensional accuracy and mechanical properties, as well as the significant disparities between manufacturer-provided values and actual measured mechanical properties, highlighting the importance of experimental verification. Hence, the findings of this study hold value not only for the scientific community but also for hobbyist printers and beginners in the 3D printing realm seeking guidance in material selection for their projects. Furthermore, the methodology employed in this research can be adapted for evaluating a broad range of other 3D printing materials.

## 1. Introduction

Additive technologies, dating back to the 1980s, have revolutionized the manufacturing industry. This unconventional manufacturing technology operates on the principle of creating a body by successively placing layers of material on top of each other [1,2]. It is used primarily for small series production and mainly to produce prototypes and concept models. The best-known and most widely used 3D printing method is FDM/FFF (fused deposition modeling/fused filament fabrication). In this method, the fused material is deposited in the form of a filament of a given diameter onto a printing substrate [3,4,5]. Due to its simplicity and low cost, this method is particularly popular with hobby printers. PLA (polylactic acid) is the most widely used printing material, and this is the main reason why the authors focused on this material [6,7,8,9,10].

**Effect of print temperature, print speed, layer height, and filament color**

Tang et al. [11] reported in their paper that an increase in printing temperature to between 200 and 230 °C increased the strength by 4.3%, but a decrease of 1.02% followed at 240 °C. Hsueh et al. [12] found that the strength of samples printed at 190 °C increased significantly compared to samples printed at 180 °C. This was followed by an almost linear increase up to a maximum value of 220 °C. However, this trend was only true for printing speeds above 40 mm/s; at 35 mm/s the samples printed at 210 °C reached the highest strength, and the difference between 190 °C and 220 °C was very small. Hsueh et al. [13] also found in another paper that increasing the printing temperature and infill percentage had a positive effect on the hardness (measurements according to Shor D). Nugroho et al. [14] conducted an experiment in which they made a total of 25 specimens (5 × 5) with five different layer heights (0.1–0.5 mm) for the bending test. The ultimate strength reached its highest value of 59.6 MPa for the largest layer height, although the values hardly differed within the range of 0.1–0.4 mm (ranging between 43.6 and 44.1 MPa). Pandzic et al. [15], in their work, investigated the effect of the filament color on the mechanical properties. They demonstrated that color additives indeed affect both strength and ductility, with differences between colors being around 31.5% for strength and a staggering 1442.9% for ductility.

**Effect of model orientation during printing and raster selection**

The orientation of the model on the print substrate and the orientation of the print “raster” are important parameters in FDM printing due to the anisotropic nature of printing and the layer-by-layer principle [16]. Nugroho et al. [14] also showed that for better ductility and toughness, a layer grid in a −45/45° orientation was used for these samples. In their research, Corapi et al. [17] found that in terms of tensile strength, the X-axis (supine) orientation was the most advantageous, achieving 9.7% higher strength than the Y-axis (lateral). The difference in ductility was equal to 3.12%, with the Y-axis proving to be more advantageous. In their next paper, Hsueh et al. [18] tested different combinations of tilts in the given directions in addition to the basic X-, Y-, and Z-orientations. Surprisingly, the samples printed at 60° to the X-axis achieved the highest strength. For the X-axis samples, a comparison of different grid orientations was also performed. The highest strength was achieved by the cross grid of −45/45°. Lim et al. [19] found that samples printed on the Y-axis achieved more than three times the strength of the Z-axis samples. Hanon et al. [20] also found in their work that the X-axis orientation was the most advantageous in terms of strength, samples printed in this orientation achieved 15.07% higher strength at 100% infill compared to the Y-axis samples and even 58.7% higher strength compared to the Z-axis samples. When only 50% infill was used, the difference between X- and Y-axis samples was only 7.6%. In terms of the raster, the 0/45° combination proved to be the most advantageous, achieving 3.36% higher strength than the −45/45° combination. Naveed [21], in his investigation on the effect of printing speed and raster orientation, concluded that for X-axis printing, with a layer height of 0.1 mm and temperature of 200 °C, the combination of −45/45° cross raster and lower printing speed, namely, 35 mm/s, was more advantageous in terms of strength. Samples with this combination of parameters achieved 9.87% higher strength compared to samples with the same raster and 65 mm/s, and 9.13% higher strength compared to samples with the same printing speed but with a 0–90° cross raster. In all cases, the samples with the −45/45° raster also achieved higher ductility compared to the samples with the 0/90° raster. Letcher [22] stated in his work that for rasters without layer alternation, the 45° orientation is the most advantageous, but in terms of ductility, it is the 90° orientation that achieved the highest accuracy of all orientations. Zhang et al. [23] reported that, in terms of both tensile strength and ductility, the single 0° grid is the most advantageous, achieving, for example, 9.66% higher strength and 7.95% higher ductility compared to the frequently used −45/45° cross grid. Lalegani Dezaki et al. [24] investigated the effect of the tilt of the specimens on the printing pad on the strength value. They concluded that the X-axis orientation of the specimen, i.e., no tilt, was the most advantageous. However, this claim is in direct contradiction to the claim made by Hsueh et al. [18]. The difference between the X- and Y-axis orientations here is more than 70%. Reverte et al. [25] also investigated the effect of print orientation on the substrate, but here the Y-axis was found to be the most favorable orientation in terms of strength when using 100% infill and a layer height of 0.16 mm, which contradicts some previous claims. The strength value for the Y-axis specimens was 55.7 MPa, which was 16.53% higher than the X-axis specimens. The difference between the Y- and Z-axis specimens was very significant, with the Z-axis specimens showing a strength value of only 11.5 MPa.

**Effect of shrinkage and dimensional accuracy**

Yaman [26] investigated the effect of shrinkage on 3D printed holes. He showed that all these holes are smaller than the nominal diameter and the degree of shrinkage can only be corrected to a certain extent. In their work, Zhou and Han [27] investigated the shrinkage rate as a function of fill percentage and print axis. They found that decreasing the percentage of fill slightly increased the shrinkage rate in all printing axes, where the X-axis achieved the lowest shrinkage, followed by the Z-axis, and prints printed on the Y-axis achieved the highest shrinkage. This may be due to the printer design, as most Cartesian 3D printers move the entire print pad as the Y-axis moves. In their work, García Plaza et al. [28] investigated the effect of combining different print speeds and layer heights on the dimensional accuracy of prints in all three coordinate axes (X, Y, Z). They concluded that for samples printed in “sideways” and “portrait” orientations, the X-axis achieved the highest accuracy, followed by the Z-axis, and the Y-axis achieved the lowest accuracy. Conversely, in the ‘flat’ orientation, the Z-axis achieved the highest accuracy, followed by the Y-axis, and the X-axis achieved the lowest accuracy. Although the effect of print speed and layer height on dimensional accuracy was not large, it can be concluded that in terms of maximizing print accuracy, in most cases it is advisable to choose a print speed lower than 50 mm/s and a layer height lower than 0.12 mm.

**PLA materials comparison issues**

The authors in Hodžić et al. [29] compared PLA materials from different manufacturers (3D Republic, Ultimaker, DevilDesign, PrimaSelect, PM Filament, and Moulded PLA) using an Ultimaker 3D printer. It was found that the maximum tensile force and tensile strength had differences of up to 17%. The yield strength had differences of up to 33%. Differences in the Young’s modulus were up to 7%, and the strain data showed differences of up to 170%. Furthermore, Matos et al. [30] investigated commercially available PLA filaments (different colors) that were physico-chemically characterized regarding their morphological, structural, and thermal properties. TG/DTG/DTA curves revealed different thermal stability as a function of the PLA filaments’ color, and DSC curves and XRD diffractograms were able to determine the crystallinity aspects of the commercial PLA filaments.

**Summary of the state of the art**

Based on a comprehensive analysis of the current state of the art, the authors of this study aimed to provide valuable insights for the scientific community and “hobby” 3D printer users by addressing the question of whether investing in a more expensive filament is justified in terms of the quality, dimensional accuracy, and mechanical properties of the 3D printed parts. The study focused on the most used material for FFF 3D printing, namely, PLA (polylactic acid), as indicated by ref. [6]. Considering the significant impact of filament color on mechanical properties [15], white-colored filaments were specifically investigated. Filament manufacturers widely available in Central Europe were selected for analysis. Previous research [11,12,13] has demonstrated that variations in printing temperature have a rapid effect on the mechanical properties of printed parts. The influence of print orientation and support structures on mechanical properties has also been explored in studies [14,17,18,19,20,21,22,23,24,25], although the findings were inconsistent and contradictory at times (e.g., [18,24]). The shrinkage and dimensional accuracy of prints were investigated in studies [26,27,28], with refs. [29,30] being the closest to the present experiment. However, ref. [29] utilized a less commonly used 2.85 mm filament diameter and an Ultimaker S5 printer, which falls outside the category of hobby 3D printers due to its price. Additionally, ref. [29] did not specify the filament color or assess the print quality and dimensional accuracy. In ref. [30], filaments of different colors were selected from the start, impacting the print properties according to [15]. Moreover, ref. [30] mainly focused on the physico-chemical properties, emphasizing morphology, structure, and thermal characteristics, which are of lesser relevance and interest to the hobbyist community, who primarily prioritize part strength, quality, and dimensional accuracy.

The aim of this work was to propose a suitable methodology that would ensure consistent conditions (same material color, printing temperature, printing orientation, filament diameter, and printer) for obtaining the most meaningful results. The proposed methodology involved a comparative analysis of commercially available PLA materials in terms of their mechanical properties, quality, and dimensional accuracy through a tensile test conducted in accordance with the ČSN EN ISO 527−1 standard. Additionally, a specially designed artifact was inspected using computed tomography based on the recommendations of ISO/ASTM 52902:2019. This study not only provides insights into the selected filaments across various aspects but also serves as a valuable methodology for assessing other materials used in FFF technology.

## 2. Materials and Methods

### 2.1. Selected Filaments

The testing aimed to compare the FFF/FDM printing material that is currently most used, namely, polylactic acid (PLA) [6]. This is a degradable biomaterial, produced most often from corn or potato starch. Its popularity is due to the ease of printing (with no significant shrinkage during cooling), low cost, and virtually zero toxicity. While it is recyclable to some extent, the gradual deterioration of its properties must be considered. As a material, it is strong, but brittle and lacks ductility. However, its biggest disadvantage is its susceptibility to degradation due to sunlight and low heat resistance. The glass transition temperature for this material ranges from 60 to 65 °C, beyond which it changes its properties and becomes softer [31].

A total of 8 manufacturers were selected for testing. These products are easily purchasable in the commercial market and are suitable for hobby printers, for example. At the same time, these filaments were chosen due to their availability throughout Europe and some of them even worldwide. The manufacturers were Prusament [32], Devil Design [33], Sunlu [34], C-Tech [35], Verbatim [36], Tronxy [37], Plasty Mladeč [38], and Gembird [39]. The price criterion was also included in the final evaluation, as these manufacturers cover a considerable price range, so this testing will show whether the price difference between the individual filaments is justified.

A uniform diameter of 1.75 mm was chosen for all the filaments, and white was chosen as the color, as it is found that color influences the mechanical properties. To minimize the wetting of the filament due to improper storage or packaging, all filaments were dried before printing using a Sunlu FilaDryer S1 filament dryer (Zhuhai SUNLU Industrial Co., Ltd., Zhuhai City, China), maintaining a constant temperature of 45 °C for 6 h [15].

### 2.2. Printer and Parameters

All samples and artifacts were printed on the Original Prusa i3 MK3S+ printer (Prusa Research a.s., Prague, Czechia), which is reasonably priced (EUR 1159) and, thus, affordable for the hobby printer community. It is also widely available. The printing plate was chosen to be a standard smooth PEI plate. The PrusaSlicer 2.3.0 (Prusa Research a.s., Prague, Czechia) program was chosen for print preparation. The testing aimed to compare different filament manufacturers using ideal basic and identical slicer settings (printing profile 0.2 mm quality) and an easily adjustable 100% infill. This choice was made based on state-of-the-art results, as it was found that refs. [11,12,13] proved that a change in printing temperature leads to a rapid change in the mechanical properties of the part, and studies [14,17,18,19,20,21,22,23,24,25] concluded that the effect of the grid and print orientation can influence the mechanical properties of the part. In addition to the basic slicer settings, the selected print temperature is also listed in the recommended temperature range in the technical sheet for all selected manufacturers. These results could act as a source of information for 3D printing beginners when choosing the right filament.

Z-axis prints showed problems in the form of misprints that often came off the mat before the print was complete. The solution was to use three layers of a raft, a printing collar (brim) with a width of 5 mm, and reduce the flow to 95%. However, to preserve the authenticity and accuracy of the measurement, it was necessary to use this reduced value of the extrusion multiplier also for the samples in the X- and Y-axes. For the samples in the Y-axis, supports were used with a modified distance from the print from 0.1 mm to 0.2 mm. To preserve the objectivity of the measurements, these changes were made for all manufacturers. The final printing parameters are shown in Table 1.

### 2.3. Tensile Test

**Figure 1:**
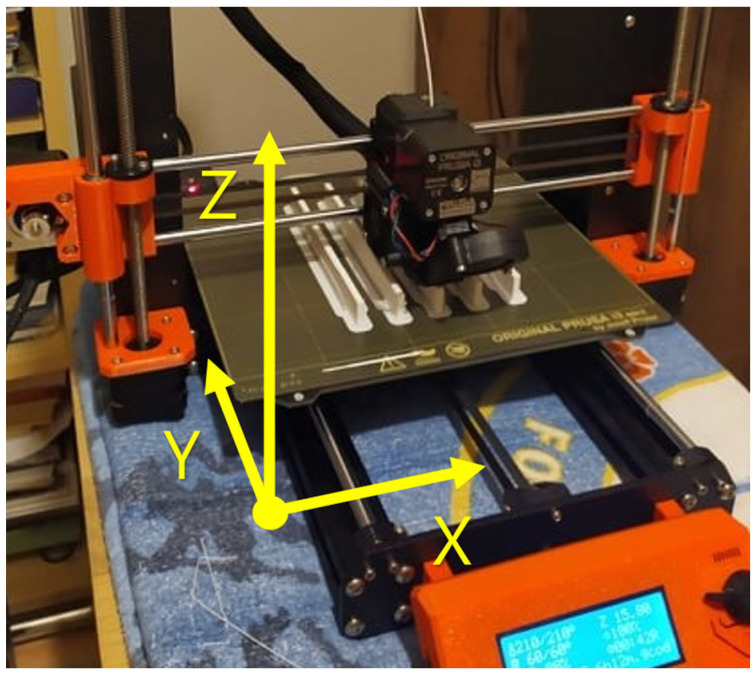
Indication of the orientation of test specimens in individual axes.

The tensile test was carried out according to CSN EN ISO 527-1 with specimens 1B according to CSN EN ISO 527-2 on a testing machine from Walter+Bai AG (Walter + Bai AG, Löhningen, Switzerland). The specimens were printed according to Figure 1 in all three orientations according to the axes (X, Y, Z). Five specimens were printed from each orientation, thus a total of 15 specimens were printed for each manufacturer.

The room temperature was maintained at 20 °C throughout the tests. A speed of 50 mm/min was chosen as the test speed. For each specimen, the thickness, h, and the width of the tapered part, b, were measured before testing using a digital caliper to determine the initial cross-section. An important aspect of the tensile testing of the plastics was the correct clamping of the specimens, as these specimens are very susceptible to slipping in the jaws. During clamping, the standard clamping distance between the jaws also had to be maintained, for specimen 1B this distance was 115 mm. The output of this test was a working tensile diagram that recorded the relationship between the loading force, F, and the elongation, ΔL, at each point in the test:

*Test specimen cross-section*:

A = b × h(1)

where b is the width of the tapered section and h is the thickness of the specimen.

*Tensile strength*:

σ_m_ = F_max_/A (2)

where F_max_ is the first maximum loading force.

*Nominal relative elongation at break*:

ε_tb_ = L_tb_/L (3)

where L_tb_ is the elongation of the test specimen before breaking and L is the clamping distance between jaws.

### 2.4. Test Artifact and CT Analysis

An analysis of all the test artifacts was performed using a Zeiss Metrotom 1500 (Carl Zeiss AG, Oberkochen, Germany)—industrial computed tomography. Industrial computed tomography is nowadays the fastest-growing field of non-destructive research. The basic principle of computed tomography is to illuminate the object under investigation with X-rays from various angles using hundreds to thousands of images. The computer software first reconstructs individual 2D planar sections that are covered with so-called voxels (volume elements), then assigns absorption coefficients to these voxels individually, thus distinguishing between multiple types of materials. It then composes a 3D spatial image from the 2D surface sections [40].

**Figure 2:**
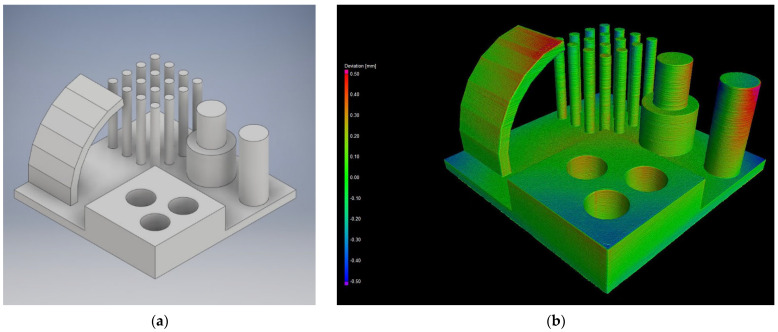
Test artifact for CT analysis. (**a**) Artifact design in CAD; (**b**) final measurements dimensional check.

The test artifact (Figure 2a) has a base plate size of 100 × 100 × 5 mm conforming to the recommendations of ISO/ASTM 52902:2019 on test artifacts for additive manufacturing. The artifact contains 4 separate parts, the first part is a print overhang test with a range of 0 to 75° and “layers” of 15°. The second part is the stringing test, consisting of sixteen narrow and tall pins. The third part is made up of two pins, the diameter of one of which is subsequently changed halfway through. These pins verify the ability to print dimensionally accurately. The last part is a block with three holes. By examining these, the degree of shrinkage for each manufacturer is determined, as well as the orientation or potential cylindricity of the holes. However, these holes do not have uniform dimensions but are offset by 0.1 mm (15.1–15.3 mm). This range serves as a test of composability, as one of the pins (15 mm diameter) is cut off and subjectively tested for composability.

The first step was to clamp the artifacts before scanning. This phase is very important because due to the principle of tomography and the 360° rotation of the sample, good clamping is necessary. Inadequate clamping will cause unwanted movement of the sample, leading to “noise” and inaccurate interpretation of the point cloud. Furthermore, the choice of clamping material is important. The CT scanner recognizes materials by their density during scanning, so a material that has a very low density is necessary and can be easily filtered out of the scanned point cloud during evaluation. For this reason, polystyrene was chosen. The samples were placed in pairs in the tomograph and the supporting polystyrene block in a “sideways” orientation and additionally supported by other smaller polystyrene blocks for extra stability.

This was followed by the scanning process itself. The scanning was carried out with the following parameters:

- voltage: 180 kV;
- current: 897 μA;
- detector resolution: 2048 × 2048 px;
- individual voxel size: 89.36 μm.

Scanning each pair of artifacts took approximately 50 min. After the scanning process (Figure 2b), the VGSTUDIO MAX 3.2.2 (Volume Graphics GmbH, Heidelberg, Germany) software was used for evaluation. This program allows users to filter out the two artifacts from the scanned point cloud, then separate them from each other and place them in space according to the three given planes using the 3–2–1 registration function. It is also possible to import the original CAD model in STEP format. This was placed in the same space using the same function as the scanned model, so it was possible to measure single-individual deviations and present them using a color map. In addition to the color map, some specific dimensions and angles were also measured, e.g., the dimensions of all pins, internal holes, angle of maximum inclination, and a large statistical set of 16 pins for the string-wrap test, which gives a good indication of the accuracy and repeatability of the print.

Due to there being many different tests, it was necessary to determine the final methodology for comparing individual manufacturers and determining the ranking. For this reason, the so-called multi-criteria evaluation of variants (VHV) was used, which is suitable for complicated decisions with the assessment of multiple aspects. The individual examined criteria had the same weight in the final evaluation. For each criterion examined, the evaluation determined the order from the best to the worst result and this ranking was scored according to a downward trend from 8 to 1 points. The points range and the system of awarding points were identical for all criteria; if the results within one criterion were equal, the same number of points were awarded to each manufacturer. After including all the criteria, the final sum was made, and the ranking was determined. However, since one of the objectives of this work was to compare producers by price, a price criterion was also added to the evaluation. The price per kilogram of filament for each manufacturer was divided by the total number of points earned and the ranking was determined according to the “price:performance” ratio, i.e., the better result was achieved by the manufacturer who needed a lower amount per point earned.

## 3. Results

### 3.1. Tests of Mechanical Properties

For the tensile test, printed samples in the X-, Y-, and Z-axes were used for comparison. The output of this measurement was the values of the ultimate tensile strength of σ_m_ and the nominal relative elongation at the break of ε_tb_. These values have been tabulated and are shown in the relevant figures.

#### Tensile Test of Test Specimens in the X-, Y-, and Z-axes

**Figure 3:**
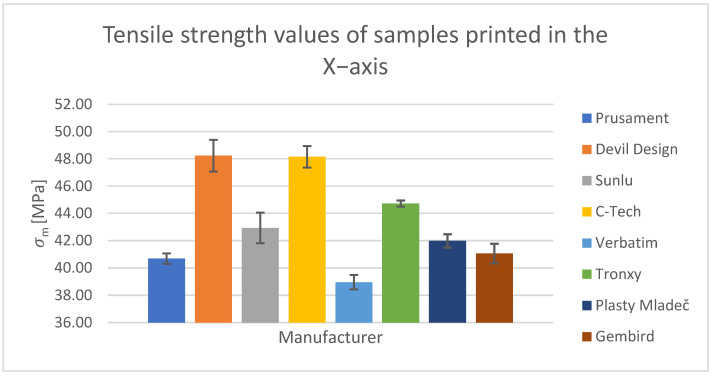
Tensile strength values of samples printed in the X-axis.

**Figure 4:**
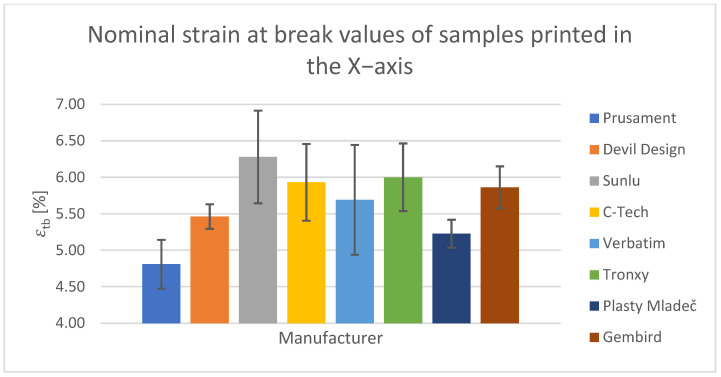
Nominal strain at break values of samples printed in the X-axis.

**Figure 5:**
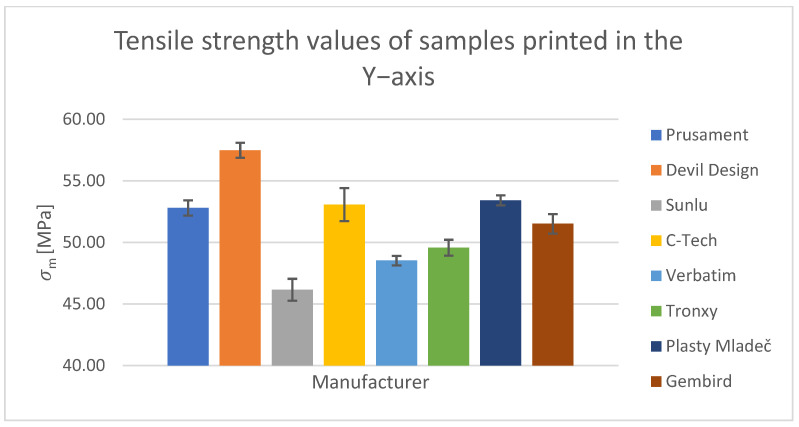
Tensile strength values of samples printed in the Y-axis.

**Figure 6:**
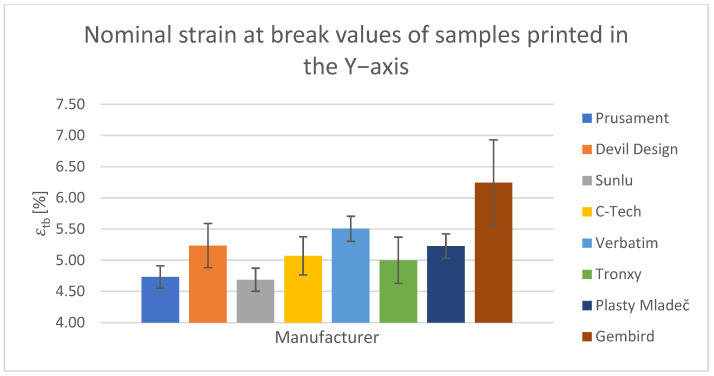
Nominal strain at break values of samples printed in the Y-axis.

**Figure 7:**
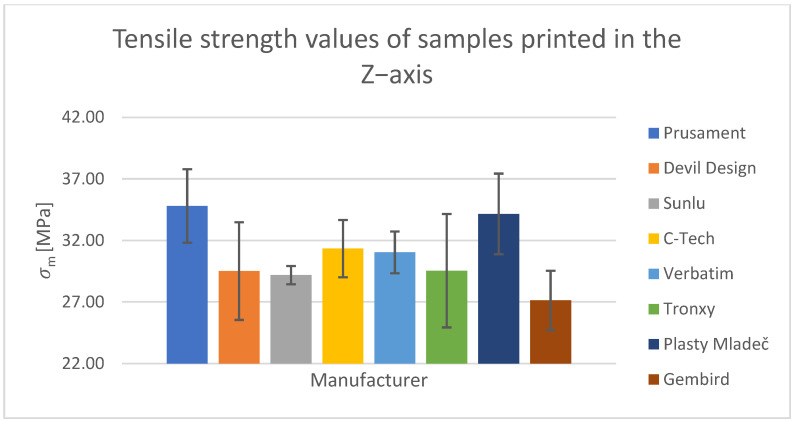
Tensile strength values of samples printed in the Z-axis.

**Figure 8:**
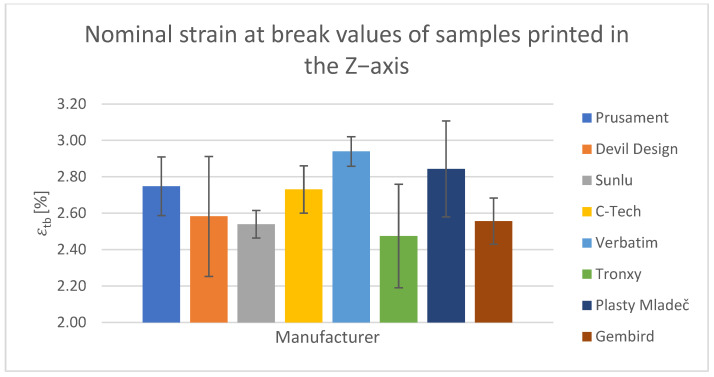
Nominal strain at break values of samples printed in the Z-axis.

The obtained test results pertain to three axes: X-axis (Figure 3 and Figure 4), Y-axis (Figure 5 and Figure 6), and Z-axis (Figure 7 and Figure 8). The tensile test, as depicted in Table 2, revealed that the optimal print orientation in terms of strength was along the Y-axis. Devil Design exhibited the highest strength, of 57.5 MPa, in this orientation. Similarly, on the X-axis, Devil Design also achieved the most favorable results, attaining a strength of 48.2 MPa. Conversely, the poorest results in terms of orientation were observed in the Z-axis, while Prusament performed the best with a strength of 34.8 MPa. These outcomes contradict the findings of previous studies [17,20,24], where the authors suggested that the X-axis orientation outperformed the Y-axis orientation in terms of strength. However, our results align with another study where samples printed along the Y-axis demonstrated over three times higher tensile strength [19].

### 3.2. Dimensional Accuracy Analysis Using CT

The dimensional accuracy analysis consisted of measuring the individual parts of the test artifact. The entire part of the test artifact was scanned. The analysis of individual parts was carried out using VGSTUDIO MAX.

#### 3.2.1. Pin Dimensional Accuracy Analysis

**Figure 9:**
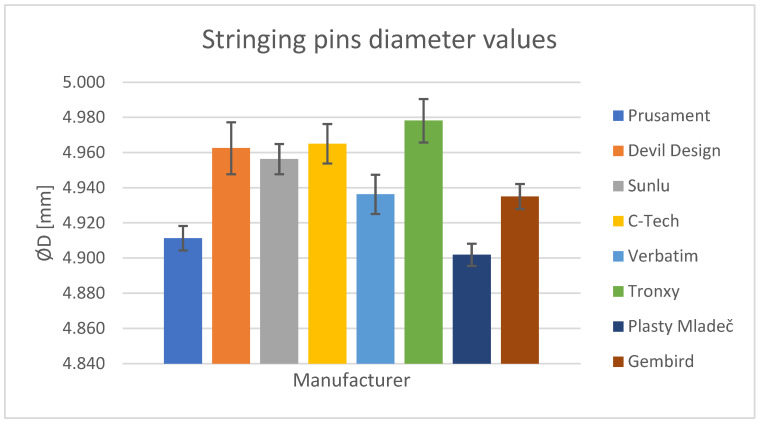
Stringing pin diameter values.

In the initial phase of analyzing the dimensional accuracy, the objective was to determine the average diameter of the pins used for stringing. Given the substantial sample size of 16 items, it was possible to accurately estimate the overall printing accuracy of the manufacturer (Figure 9) based on this dataset.

Among the manufacturers, Tronxy (Table 3) exhibited the closest diameter to the nominal value of 5 mm, measuring 4.978 mm. Conversely, Plasty Mladeč had the largest deviation from the nominal size, with a diameter of 4.902 mm. Nevertheless, the difference between the highest and lowest recorded values was relatively insignificant at 1.55%. The results indicate that all dimensions were smaller than the nominal diameter, with an average deviation of 1.14%.

#### 3.2.2. Analysis of Dimensional Accuracy of Pins

**Figure 10:**
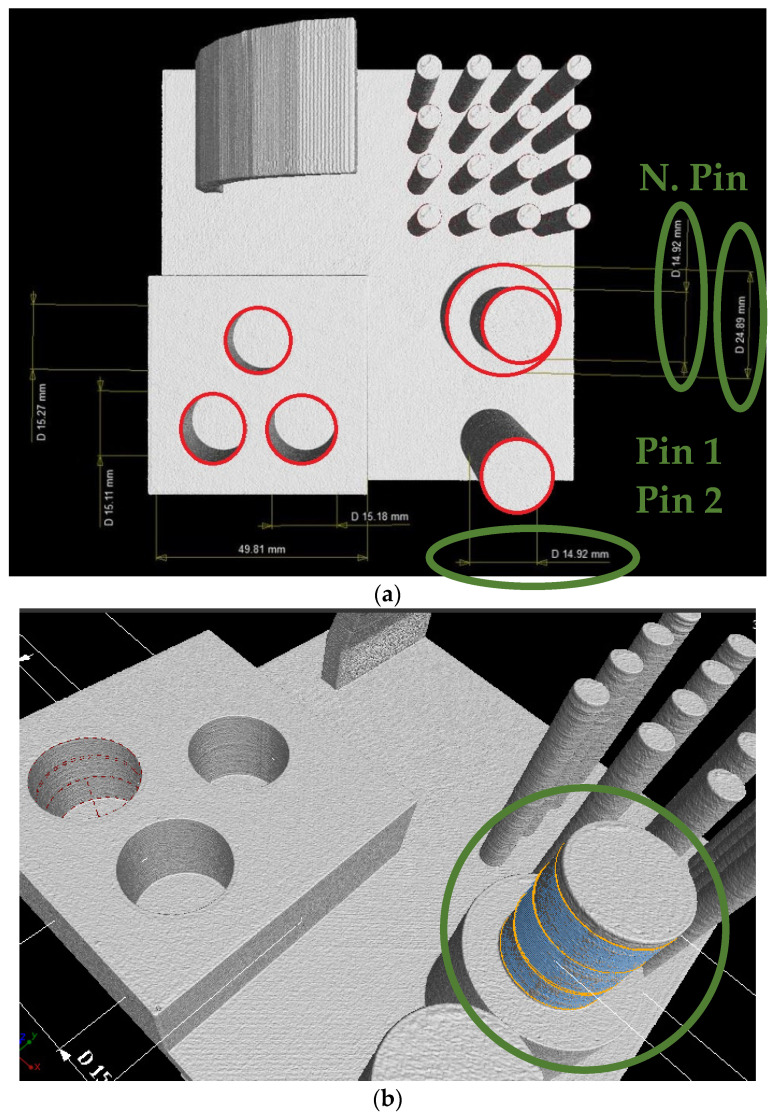
(**a**) Measured dimensions of pins and internal holes (Verbatim, marked in red). (**b**) Statistical evaluation with form deviation check.

During the examination of the pin dimensions (Figure 10), a noticeable pattern emerged: all pins displayed dimensions smaller than their respective nominal diameters. Furthermore, it became evident that for pins with the same nominal dimension, the narrower pins and the narrowed section of the wider pins exhibited slight variations, with a maximum difference of 0.02 mm among all manufacturers except one. The greater deviation observed in Plasty Mladeč could be attributed to factors such as increased shrinkage or potential inaccuracies during the scanning process for diameter measurement. Consequently, it can be concluded that the stacking of objects with different diameters has minimal impact on the final diameter. Additionally, it can be observed that as the pin diameter increases, the deviation from the nominal value also increases, albeit still within hundredths of millimeters. The manufacturer Devil Design achieved the most precise diameter for the wider pin, Tronxy for the narrower section, and Devil Design again for the narrower pin. However, the maximum difference between manufacturers amounted to only 0.4% (Table 4). On average, a correlation can be observed, wherein larger pin diameters result in improved dimensional accuracy of the printed objects and slight shrinkage. For instance, with 5 mm pins, the difference from the nominal dimension is 1.14%; for 15 mm pins, the difference is 0.55%; and for 25 mm pins, the difference is 0.46%. This phenomenon may be attributed to material characteristics or variations in cooling during the printing process which affect individual pins at different times and to varying extents, despite consistent cooling intensity.

#### 3.2.3. Analysis of Dimensional Accuracy of 3D Printed Holes

The measurements conducted on the inner hole diameters revealed a characteristic property of PLA, namely, its minimal shrinkage. The largest deviation from the nominal dimension was a mere 0.06 mm; however, there is no consistent trend indicating that all dimensions are smaller than the nominal values (Table 5). Among the four manufacturers, the measurement of the smallest holes showed a dimension that was one-hundredth of a millimeter larger than the nominal value. These findings neither confirm nor refute the results of a previous study [26], where all holes were smaller than the nominal diameter. The results indicate that Devil Design performed the least accurately for all three holes, with a deviation of 0.06 mm. In contrast, Prusament and Plasty Mladeč demonstrated the highest level of accuracy, with a deviation of only 0.01 mm each. In the analysis of hole dimensions, it can be confirmed that as the hole diameter increases, the deviation from the nominal value also increases, albeit at almost negligible magnitudes. For instance, with a diameter of Ø 15.1 mm, the average difference from the nominal value was 0.02%; for Ø 15.2 mm, the difference was 0.16%; and for Ø 15.3 mm, the difference was 0.18%.

#### 3.2.4. Analysis of Dimensional Accuracy of Pin Heights

In previous measurements, the dimensions of various parts of the artifact were examined in the X- and Y-axes. However, it is equally important to analyze the dimensions of the pins in the Z-axis. This analysis (Table 6) revealed that all samples exhibited positive deviations, indicating larger dimensions compared to the nominal values.

**Figure 11:**
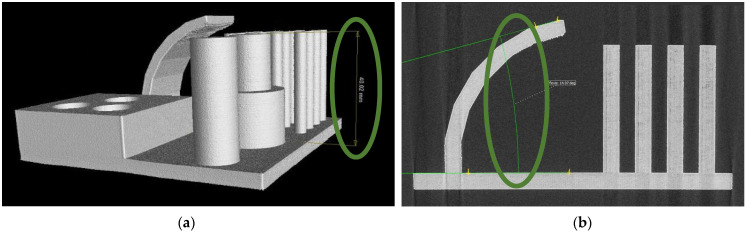
Dimensions check on CT. (**a**) Pin height (Plasty Mladeč); (**b**) overhang angle measurement (Gembird).

Nevertheless, the deviations observed in the Z-axis were even smaller than those found in the internal openings. The maximum deviation was 0.05 mm for Tronxy, while the minimum deviations of 0.02 mm were observed for Devil Design and Plasty Mladeč, resulting in a difference of only 0.075%. Figure 11a displays the artifact from the manufacturer Plasty Mladeč with measured value of pin height in green circle.

While the XY direction measurements demonstrated parts shrinking, the analysis of height accuracy in the Z-axis indicated that the printed pins were larger by 0.08% (almost identical to the nominal size). This can be attributed to the fundamental principle of 3D printing, where the model is divided into individual layers that are printed successively. As a result, the positions of these layers in the Z-axis are more precisely defined, and the effect of shrinkage is not as pronounced.

#### 3.2.5. Analysis of Printing Angular Overhangs

The final analysis conducted to assess the dimensional accuracy in printing involved measuring the angle of the largest overhang (Table 7). This measurement entailed determining the angle between the outer surface of the 75° overhang and the parallel plane of the base plate. The measured value was then incorporated into the following formula (γ = 90 − x), where γ represents the overhang angle for the plane perpendicular to the plate (ideally 75°) and x denotes our measured angle (Figure 11b).

The manufacturer C-Tech achieved the best result, with an overhang angle precisely equal to the nominal dimension of 75°. Conversely, the largest deviation was observed in the overhang from Sunlu, with a deviation of 0.88°. Consequently, due to the perpendicular plane, the printed overhang resulted in an angle of 74.12°. It should be noted that deviations in this measurement are not exclusively positive or negative. The complexity of the printing process, along with several factors and to some extent chance, influence the results. Furthermore, it is important to acknowledge that the quality and accuracy of the surface diminish as the overhang angle increases, thereby impacting the result and repeatability of the measurement. However, this effect becomes noticeable only for overhang angles exceeding 45°.

### 3.3. Print Quality Analysis

The evaluation of print quality was conducted using subjective ratings (Table 8). The assessment encompassed various aspects, including the degree of stringing, surface quality of the lower portion of overhangs, and overall assembly. The results are presented in the table below.

**Figure 12:**
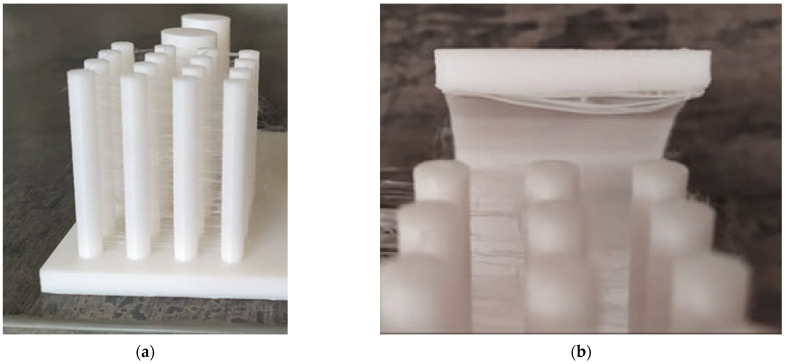
Print quality analysis. (**a**) Stringing (Sunlu); (**b**) saggy 75° overhang (Sunlu).

Prusament and Plasty Mladeč achieved the best results in terms of stringing rates (Figure 12a), with both manufacturers essentially exhibiting no stringing issues. Devil Design, Gembird, and Verbatim had slight instances of stringing, but still within acceptable limits. On the other hand, Tronxy and C-Tech exhibited significant stringing, while Sunlu recorded the worst results, with its 16 pins covered by dense networks of “webs”. The problem of stringing was not limited to the pins but also appeared during transitions from pins to overhangs. One of the pins displaying stringing can be observed in the provided image.

The subjective evaluation of overhang surface quality, as shown in Figure 12b, proved to be complex. Most manufacturers achieved fairly similar results, but upon considering factors such as excess material accumulation, visible sagging, and inconsistencies in individual layer lines, C-Tech and Tronxy emerged as the top performers. Prusament, Verbatim, and Gembird also attained very good results, although slight inconsistencies in layer deposition and more pronounced material accumulation in peripheral areas of the overhangs influenced their rankings. Sunlu exhibited the best surface quality among all the manufacturers when printing a 60° overhang. However, significant sagging and surface degradation were evident at a 75° overhang, as clearly depicted in Figure 12b.

**Figure 13:**
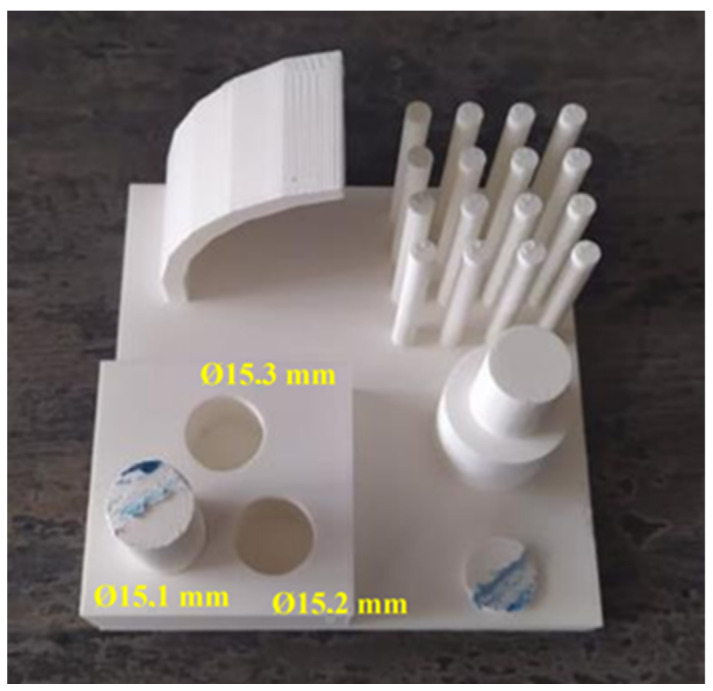
Demonstration of composability of an artifact (Plasty Mladeč).

During the composability test (Figure 13), Prusament and Plasty Mladeč emerged as the top performers. They achieved the best scores as the pins effortlessly fit into even the smallest hole with a diameter of 15.1 mm (Figure 13). On the other hand, Tronxy demonstrated the poorest performance, successfully assembling only with a hole diameter of 15.3 mm. For all other manufacturers, optimal assembly was achieved with a hole diameter of 15.2 mm, but it required applying greater forces. Notably, some manufacturers, such as Gembird, found the diameter of 15.15 mm to be the optimal size for the hole during the testing process.

## 4. Discussion

To comprehensively determine the top PLA manufacturer, a final table (Table 9) was created considering the significant variations observed across the different tests conducted. The evaluation criterion involved assigning scores based on the ranking of individual tests, with the best manufacturer receiving eight points and subsequent positions receiving one point less, while the worst manufacturer received only one point. However, due to notable standard deviations, the measurement of nominal relative elongations at break was excluded from this assessment. This value holds relatively less importance for the average user in most cases, as it primarily indicates the material’s fragility, whereas dimensional accuracy and strength are more desirable qualities. It is important to note that the scores for stringing, overhang quality, and composability were assigned based on the subjective evaluation of the authors [41].

According to the adopted methodology, the results indicate that the PLA filament from C-Tech performed exceptionally well, closely followed by Plasty Mladeč and Devil Design. On the other hand, Sunlu exhibited the poorest performance among the tested manufacturers. Considering the price factor, it is evident that C-Tech’s PLA filament stands out as the optimal choice. It is clear that the price does not necessarily correlate with the quality of the end product. Despite being the least expensive option, Sunlu yielded the worst results and ranked second to last even in terms of price evaluation. Conversely, C-Tech, being the second cheapest, emerged as the top choice overall. Verbatim’s PLA, despite its significantly higher cost, demonstrated that the most expensive filament does not always guarantee the best performance. It consistently achieved average to below-average results across all tests, failing to justify its premium price tag. For users prioritizing mechanical properties, Devil Design and C-Tech are the recommended choices. Conversely, users seeking high print quality and aesthetic appeal should opt for Prusament or Gembird.

Upon comparing the measured strengths with the manufacturer’s data presented in Table 10, notable discrepancies become apparent in certain cases. Devil Design and Plasty Mladeč closely align with the stated values, while Sunlu, Verbatim, and Tronxy exhibit significant deviations from these values. For instance, Verbatim’s measured value is 14.5 MPa lower than the stated value. It should be noted that some manufacturers do not provide any information regarding tensile strength. Although certain manufacturers, such as Sunlu or Tronxy, furnish users with these values, they often fail to specify the testing conditions or the methodology employed, warranting caution when interpreting such data. In contrast, manufacturers such as Prusament or Verbatim include precise details regarding the test conditions and standards used in their datasheets. This inconsistency served as one of the motivations for conducting this research.

The evaluation has revealed several intriguing findings. Firstly, the dimensional accuracy of the pins and the mechanical properties, influenced by the print orientation, directly contradict the hypotheses presented in the research. Syrlybayev et al. [42] posit that all deviations in external dimensions are in the positive direction, indicating an increase in the actual value. However, in our experiment, all deviations were in the negative direction. Additionally, a correlation has been observed, indicating that the dimensional accuracy of printouts improves while the printout shrinks as the diameter of the pins increases (5 mm pins differ by 1.14% from the nominal size; 15 mm pins differ by 0.55%; 25 mm pins differ by 0.46%). The dimensional analysis of holes further confirms that as the hole diameter increases, the deviation from the nominal value also increases, albeit insignificantly (Ø 15.1 mm exhibited a mean difference of 0.02% from the nominal value; Ø 15.2 mm had a difference of 0.16%; Ø 15.3 mm had a difference of 0.18%).

**Figure 14:**
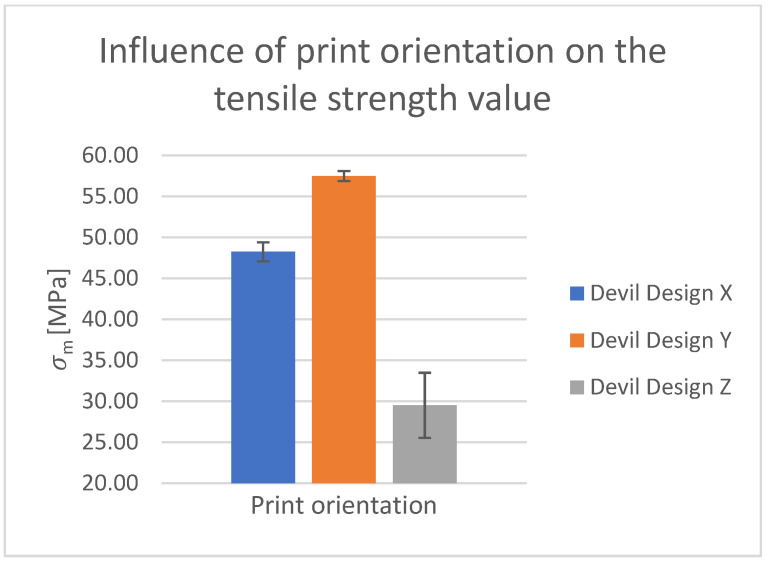
Influence of print orientation on the tensile strength value.

Regarding the accuracy of the Z-axis height, it was discovered that the printed pins were larger by 0.08%, which is nearly identical to the nominal size. Conversely, Hanon et al. [20] suggest that the X-axis offers the best printing orientation in terms of strength. However, our measurements conducted in this study demonstrated the Y-axis as the most advantageous orientation for all manufacturers. For instance, for Plasty Mladeč there is a difference of over 27% between these orientations. Nevertheless, the hypothesis regarding the Z-axis samples was validated, as these samples exhibited the lowest strength since the fibers are essentially torn apart due to their orientation in the tensile test. It requires significantly less force to separate them compared to breaking the perimeters parallel to the orientation of the testing machine and the 45°-angled infill observed in samples printed in the Y-axis. The distinction between the X- and Y-axis samples can also be attributed to the varying quantity of perimeters oriented parallel to the tearing machine’s feed. In the X-axis, the perimeters contribute to the specimen’s thickness, whereas in the Y-axis, the entirety of the test specimen’s width is composed of perimeters due to the sideways orientation. The comparison between the orientations is depicted in Figure 14 [20].

## 5. Conclusions

The objective of this article was to propose a suitable methodology to compare commercially available PLA materials and determine whether a more expensive filament positively influences the mechanical properties, quality, and dimensional accuracy of 3D printed parts. The uniqueness of the study lies in following consistent conditions, including the same material color, printing temperature, printing orientation, filament diameter, and printer, to yield the most meaningful results. The detailed presentation and discussion of the results can be found in the Discussion section. Key observations derived from the results are as follows:

- Contrary to the common assumption that price indicates quality, the study revealed that the second-cheapest PLA achieved the best overall results within the employed methodology.
- Discrepancies of up to 20% were observed between the declared values provided by manufacturers and the actual measured values of mechanical properties, highlighting the importance of experimental verification.
- The orientation along the Y-axis demonstrated the highest strength, while the Z-axis orientation yielded the poorest results.
- All measured pin dimensions were smaller than the nominal diameters.
- A correlation was observed, indicating that as the pin diameter increased, the dimensional accuracy of the printed parts improved (e.g., for 5 mm pins, the difference was 1.14% from the nominal dimension; for 15 mm pins, the difference was 0.55%; for 25 mm pins, the difference was 0.46%).
- The deviation from the nominal value increased with larger hole diameters (e.g., for Ø 15.1 mm, the mean difference was 0.02% from the nominal value; for Ø 15.2 mm, the difference was 0.16%; for Ø 15.3 mm, the difference was 0.18%).

The results of this study hold significance not only for the scientific community but also for hobby printers and beginners in 3D printing who seek guidance in selecting the best material for their projects. Furthermore, the methodology employed in this research can be applied to a wide range of other 3D printing materials.

## Figures and Tables

**Table 1:** Printing parameters.

Parameters	Value
Nozzle temperature	210 °C
Heatbed temperature	60 °C
Layer height	0.2 mm
Infill print speed	80 mm/s
Inner perimeters print speed	45 mm/s
Outer perimeters print speed	25 mm/s
Top layers print speed	40 mm/s
Infill	100% (raster −45:45)
Extrusion multiplier	0.95 (100 ^1^)
Printing orientation	0° (parallel to Y)
Artifact print time	10 h 25 min
X-axis sample print time	3 h 33 min
Y-axis sample print time	6 h 12 min
Z-axis sample print time	6 h 38 min

^1^ Value used for artifact.

**Table 2:** Results of tensile test for samples printed in the X-, Y-, and Z-axes.

Orientation	X-Axis		Y-Axis		Z-Axis	
Manufacturer	σ_m_ (MPa)	Ɛ_tb_ (%)	σ_m_ (MPa)	Ɛ_tb_ (%)	σ_m_ (MPa)	Ɛ_tb_ (%)
Prusament	40.7 ± 0.4	4.81 ± 0.33	52.8 ± 0.6	4.73 ± 0.18	34.8 ± 3.0	2.75 ± 0.16
Devil Design	48.2 ± 1.2	5.46 ± 0.17	57.5 ± 0.6	5.23 ± 0.35	29.5 ± 4.0	2.58 ± 0.33
Sunlu	43.0 ± 1.1	6.28 ± 0.64	46.2 ± 0.9	4.69 ± 0.19	29.2 ± 0.7	2.54 ± 0.08
C-Tech	48.2 ± 0.8	5.93 ± 0.53	53.1 ± 1.3	5.07 ± 0.31	31.3 ± 2.3	2.73 ± 0.13
Verbatim	39.0 ± 0.5	5.69 ± 0.75	48.5 ± 0.4	5.50 ± 0.20	31.0 ± 1.7	2.94 ± 0.08
Tronxy	44.7 ± 0.2	6.00 ± 0.46	49.6 ± 0.6	5.00 ± 0.37	29.5 ± 4.6	2.47 ± 0.28
Plasty Mladeč	42.0 ± 0.5	5.23 ± 0.19	53.4 ± 0.4	5.23 ± 0.20	34.2 ± 3.3	2.84 ± 0.26
Gembird	41.1 ± 0.7	5.86 ± 0.29	51.5 ± 0.8	6.24 ± 0.69	27.1 ± 2.4	2.56 ± 0.13

**Table 3:** Stringing pin diameter values.

Manufacturer	Diameter (mm)
Prusament	4.911 ± 0.007
Devil Design	4.963 ± 0.015
Sunlu	4.956 ± 0.009
C-Tech	4.965 ± 0.011
Verbatim	4.936 ± 0.011
Tronxy	4.978 ± 0.012
Plasty Mladeč	4.902 ± 0.006
Gembird	4.935 ± 0.007

**Table 4:** Pin diameter values.

Manufacturer	Pin 1 Diameter (mm)	Narrowed Pin Diameter (mm)	Pin 2 Diameter (mm)
Prusament	24.88 ± 0.07	14.91 ± 0.01	14.90 ± 0.05
Devil Design	24.91 ± 0.08	14.93 ± 0.05	14.94 ± 0.07
Sunlu	24.86 ± 0.02	14.89 ± 0.04	14.90 ± 0.14
C-Tech	24.88 ± 0.12	14.93 ± 0.07	14.92 ± 0.04
Verbatim	24.89 ± 0.04	14.92 ± 0.01	14.92 ± 0.01
Tronxy	24.89 ± 0.03	14.94 ± 0.02	14.93 ± 0.12
Plasty Mladeč	24.87 ± 0.05	14.93 ± 0.04	14.88 ± 0.08
Gembird	24.90 ± 0.01	14.93 ± 0.01	14.91 ± 0.17

**Table 5:** Internal holes diameter values.

Measured Hole	Hole Ø 15.1 mm	Hole Ø 15.2 mm	Hole Ø 15.3 mm
Manufacturer	Diameter (mm)	Diameter (mm)	Diameter (mm)
Prusament	15.11 ± 0.02	15.19 ± 0.01	15.29 ± 0.01
Devil Design	15.06 ± 0.04	15.14 ± 0.05	15.24 ± 0.08
Sunlu	15.11 ± 0.10	15.19 ± 0.16	15.29 ± 0.02
C-Tech	15.09 ± 0.06	15.18 ± 0.01	15.27 ± 0.01
Verbatim	15.11 ± 0.02	15.18 ± 0.27	15.27 ± 0.01
Tronxy	15.09 ± 0.08	15.18 ± 0.02	15.26 ± 0.01
Plasty Mladeč	15.11 ± 0.01	15.19 ± 0.06	15.29 ± 0.01
Gembird	15.09 ± 0.01	15.16 ± 0.03	15.27 ± 0.05

**Table 6:** Pin height values.

Manufacturer	Ø Pin Height (mm)
Prusament	40.03
Devil Design	40.02
Sunlu	40.03
C-Tech	40.04
Verbatim	40.03
Tronxy	40.05
Plasty Mladeč	40.02
Gembird	40.03

**Table 7:** Overhang angle values.

Manufacturer	Overhang Angle (°)
Prusament	15.14
Devil Design	14.84
Sunlu	15.88
C-Tech	15.00
Verbatim	15.17
Tronxy	15.21
Plasty Mladeč	14.88
Gembird	14.97

**Table 8:** Print quality analysis results.

Manufacturer	Stringing	Surface Quality of Overhangs	Composability
Prusament	8	5	8
Devil Design	6	3	2
Sunlu	1	1	5
C-Tech	3	8	3
Verbatim	4	6	4
Tronxy	2	7	1
Plasty Mladeč	7	2	7
Gembird	5	4	6

**Table 9:** Evaluation of results.

Test/Manufacturer	Prusament	Devil Design	Sunlu	C-Tech	Verbatim	Tronxy	Plasty Mladeč	Gembird
σ_m_—wires	3	8	1	7	2	5	6	4
σ_m_ X-axis	2	8	5	7	1	6	4	3
σ_m_ Y-axis	5	8	1	6	2	3	7	4
σ_m_ Z-axis	8	3	2	6	5	4	7	1
Stringing	8	6	1	3	4	2	7	5
Overhang quality	5	3	1	8	6	7	2	4
Composability	8	2	5	3	4	1	7	6
Stringing pins	2	6	5	7	4	8	1	3
Pins	3	8	1	5	5	7	2	6
Holes	8	1	8	5	5	3	8	2
Pin height	6	8	6	2	6	1	8	6
Overhang angle	5	4	1	8	3	2	6	7
Sum	63	65	37	67	47	49	65	51
**Ranks**	**4**	**2**	**8**	**1**	**7**	**6**	**2**	**5**
Price ^1^ (EUR)	23.44	19.95	14.12	16.20	24.69	18.00	23.44	17.25
Price (EUR)/point	0.372	0.305	0.383	0.243	0.524	0.368	0.360	0.336
**Ranks price/point**	**6**	**2**	**7**	**1**	**8**	**5**	**4**	**3**

^1^ Prices valid for Q3/2022.

**Table 10:** Comparison of declared and measured strengths.

Manufacturer Nominal Ø 1.75 mm	Color	Declared σ_m_ (MPa)	Measured σ_m_ (MPa)	Measured versus Declared σ_m_ (%)
Prusament [32]	Vanilla white	59.3	52.8 ± 0.6	−11.0
Devil Design [33]	White	59	57.5 ± 0.6	−2.6
Sunlu [34]	White	58.8	46.2 ± 0.9	−21.5
C-Tech [35]	White	Not specified	53.1 ± 1.3	Not specified
Verbatim [36]	White	63	48.5 ± 0.4	−23.0
Tronxy [37]	White	60	49.6 ± 0.6	−17.4
Plasty Mladeč [38]	White	53	53.4 ± 0.4	+0.8
Gembird [39]	White	Not specified	51.5 ± 0.8	Not specified

## Data Availability

Data is contained within the article.
